# Lysine lactylation in neutrophil functional remodeling across diverse disease contexts

**DOI:** 10.3389/fimmu.2026.1892562

**Published:** 2026-09-30

**Authors:** Yushu Wang, Xiaotong Liu, Chao Wang

**Affiliations:** 1 Department of Otorhinolaryngology, Head and Neck Surgery, The Second Affiliated Hospital of Harbin Medical University, Harbin, China; 2 Harbin Medical University, Harbin, China

**Keywords:** immunometabolism, lysine lactylation, neutrophil extracellular traps (NETs), neutrophils, post-translational modifications

## Abstract

Lactate, traditionally regarded as a product of glycolytic metabolism, is increasingly recognized as a metabolite that can influence cellular signaling and protein post-translational modification, including lysine lactylation (Kla). Neutrophils rely on glycolytic metabolism to support rapid effector functions and encounter substantial metabolic changes during inflammation, infection, ischemic injury, and tumor progression. Emerging studies have implicated histone and non-histone lactylation in the regulation of neutrophil-associated processes, including migration, inflammatory activation, neutrophil extracellular trap (NET) formation, and functional phenotypic remodeling. However, the extent to which these effects are mediated directly by lactylation within neutrophils remains incompletely defined, as some available evidence derives from other cell types, tissue compartments, or indirect associations with neutrophil responses. In this review, we summarize current knowledge of the molecular mechanisms regulating lysine lactylation, with a focus on lactyltransferases, delactylases, and lactylation-associated reader proteins. We further examine evidence linking lactylation-associated signaling to neutrophil migration, NET formation, and functional heterogeneity across inflammatory, ischemic, and tumor-associated settings, with consideration of distinct neutrophil populations. We also discuss the potential interplay between lactylation and other lysine acylation modifications, as well as current methodological challenges in accurately detecting and quantifying Kla and distinguishing enzymatic from non-enzymatic lactylation. Finally, we evaluate emerging pharmacological and biomarker-based approaches targeting lactate metabolism and lactylation-associated pathways. Although these studies provide a growing framework for understanding how metabolic alterations may influence neutrophil function, most evidence remains preclinical or indirect, and the relative contribution of lactylation to neutrophil responses across different pathological contexts remains to be established. Further studies using site-specific quantitative approaches, primary human neutrophils, and neutrophil-specific genetic or mechanistic models will be required to define the causal and context-dependent roles of lysine lactylation in neutrophil biology.

## 1 Introduction

Immune cells continuously adapt their metabolic programs in response to dynamic microenvironmental cues, and this process has emerged as a central mechanism regulating immune cell fate and function (1). Among the major metabolic pathways involved in immune regulation, glycolytic reprogramming plays a particularly important role during inflammation, infection, tissue injury, and tumor progression (2–4). Lactate, once regarded primarily as a metabolic end product of glycolysis, is now recognized as a metabolite with broader signaling and regulatory functions (5). The identification of lysine lactylation (Kla) further expanded the potential links between cellular metabolism and protein regulation by demonstrating that lactate-associated metabolic states can be connected to post-translational modification of histone and non-histone proteins (6, 7).

Neutrophils are the most abundant circulating leukocytes in humans and are essential components of innate immunity. Glycolysis is a major metabolic pathway supporting neutrophil effector functions, including phagocytosis, degranulation, cytokine production, and neutrophil extracellular trap (NET) formation (8–12). Changes in glycolytic activity and lactate metabolism may therefore influence the metabolic environment in which neutrophils function. However, whether these metabolic changes directly alter neutrophil function through lysine lactylation remains incompletely understood. Although Kla has been extensively investigated in macrophages and tumor cells, evidence regarding its roles in neutrophils is comparatively limited and heterogeneous. Emerging studies have associated lactylation with neutrophil migration, NET formation, inflammatory responses, and phenotypic remodeling in different pathological settings. Importantly, however, the available evidence does not always demonstrate a direct causal relationship between lactylation and neutrophil function, and some mechanistic insights are derived from other cell types or tissue compartments.

In this review, we summarize current knowledge of the molecular mechanisms underlying lysine lactylation, including enzymatic and non-enzymatic pathways, lactylation writers and delactylases, and emerging reader proteins. We then critically evaluate evidence linking lactylation to neutrophil functional responses across inflammatory diseases, ischemic injury, and cancer, with particular attention to NET formation and neutrophil heterogeneity. We distinguish findings obtained directly from neutrophils from those inferred from other cellular or experimental systems and highlight areas in which the underlying mechanisms remain uncertain. Finally, we discuss current methodological challenges and the therapeutic and translational implications of targeting lactate metabolism or lactylation-associated pathways (**Figure 1**).

**Figure 1:**
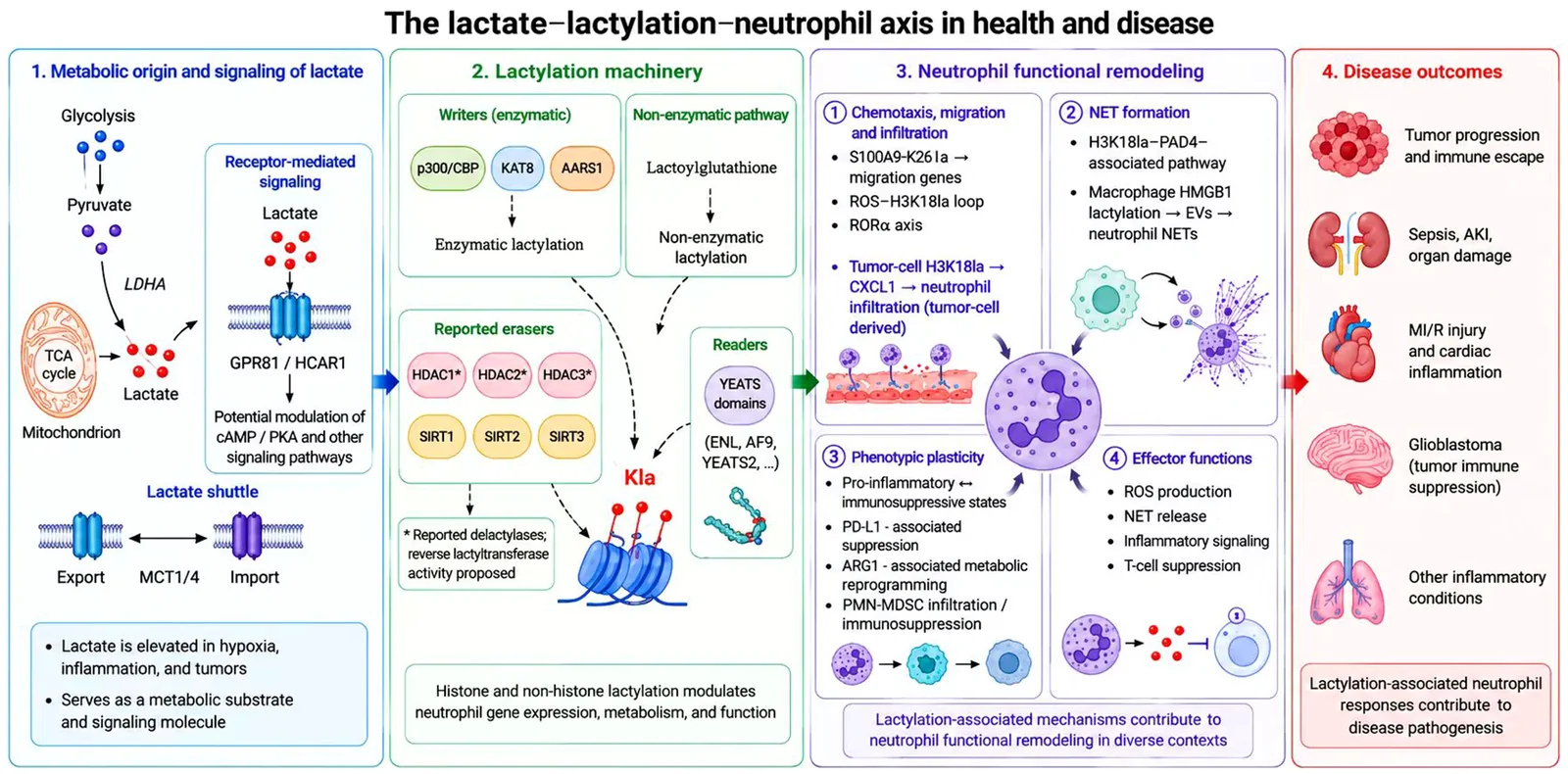
The lactate-lactylation-neutrophil axis in health and disease.

## 2 Lactate: from metabolic byproduct to signaling metabolite

Lactate was historically regarded primarily as a product of glycolytic metabolism and a contributor to tissue acidosis. Increasing evidence, however, indicates that lactate can also function as a metabolic substrate and signaling molecule under physiological and pathological conditions (13, 14). During hypoxia, inflammation, immune activation, and tumor progression, increased glycolytic flux promotes the conversion of pyruvate to lactate by lactate dehydrogenase A (LDHA) (5). Rather than representing a terminal metabolic product, lactate can be exchanged between cells and tissues and participate in metabolic adaptation within complex microenvironments.

Lactate is transported across cellular membranes mainly through proton-linked monocarboxylate transporters (MCTs), including MCT1–4 (15, 16). This transport contributes to intercellular lactate exchange and the broader concept of the lactate shuttle, whereby lactate can be taken up and reutilized as an oxidative substrate or metabolic intermediate by neighboring cells (17, 18). The biological effects of lactate therefore depend not only on its extracellular concentration but also on its cellular transport, utilization, and local metabolic context.

Lactate can also function as an extracellular signaling molecule through the G-protein-coupled receptor GPR81, also known as hydroxycarboxylic acid receptor 1 (HCAR1) (19, 20). GPR81 activation generally reduces intracellular cyclic AMP (cAMP) signaling and can influence downstream cellular responses (21–23). In neutrophil biology, available evidence supports an indirect, paracrine role for this pathway. Inflammatory stimulation increases lactate release from bone marrow neutrophils, and the released lactate acts on GPR81-expressing bone marrow endothelial cells, reducing VE-cadherin expression and increasing vascular permeability to facilitate neutrophil mobilization (24). Importantly, this study does not establish GPR81 as a neutrophil-autonomous receptor pathway. Rather, it provides evidence for metabolic communication between lactate-producing neutrophils and the surrounding bone marrow vascular compartment.

Lactate signaling through GPR81 should be distinguished from lysine lactylation (Kla). GPR81 is a cell-surface receptor that senses extracellular lactate, whereas Kla is an intracellular post-translational modification involving the covalent attachment of a lactyl group to lysine residues (6, 25). Although both processes are influenced by lactate metabolism, a direct functional connection between GPR81–cAMP signaling and histone or non-histone lactylation has not been established in neutrophils. Therefore, GPR81 is best considered a parallel lactate-sensing pathway rather than a demonstrated upstream regulator of neutrophil lactylation.

## 3 Molecular mechanisms and regulatory landscape of lysine lactylation

Lysine lactylation is a post-translational modification characterized by the covalent addition of a lactyl group to lysine residues on histone and non-histone proteins (25–29). Since its initial description as a histone modification linking glycolytic metabolism to gene regulation, Kla has been identified in a broad range of cellular and pathological contexts (6). Current evidence indicates that Kla can arise through both enzyme-mediated and non-enzymatic mechanisms and can be dynamically regulated by delactylases and potentially recognized by acylation reader proteins.

### 3.1 Enzymatic and non-enzymatic mechanisms of lysine lactylation

Lysine lactylation (Kla) can arise through both enzyme-mediated and non-enzymatic mechanisms, which differ in their chemical donors, metabolic requirements, and regulatory properties. Enzyme-mediated lactylation involves the transfer of a lactyl group from an activated donor to a lysine residue. The histone acetyltransferase p300 was among the first enzymes shown to catalyze histone lactylation, using lactyl-CoA as an acyl donor (6, 29, 30). Subsequent studies have identified additional enzymes with lactyltransferase activity in specific biological contexts, including lysine acetyltransferase 8 (KAT8) and alanyl-tRNA synthetase 1 (AARS1) (31–33). KAT8-mediated lactylation has been linked to eEF1A2-dependent protein synthesis in colorectal cancer, whereas AARS1 has been reported to function as a lactate-responsive lactyltransferase in tumor-associated contexts (31–33). However, most mechanistic studies identifying p300, KAT8, or AARS1 as lactyltransferases have been performed in cancer or other non-neutrophil systems. Therefore, these findings establish the capacity of these enzymes to mediate lactylation but do not demonstrate that they make equivalent or dominant contributions to Kla in neutrophils.

In parallel, Kla can also occur through non-enzymatic chemical acylation. Increased glycolytic flux can enhance the generation of the reactive glycolytic by-product methylglyoxal, which is converted to lactoylglutathione through the glyoxalase pathway. Lactoylglutathione can subsequently transfer its lactyl group to lysine residues through a non-enzymatic acyl-transfer reaction, without requiring a dedicated lactyltransferase (34–36). This pathway is biochemically distinct from enzyme-mediated lactylation because it relies on the spontaneous reactivity of a metabolic intermediate rather than enzyme-catalyzed transfer of a lactyl group from an activated donor. These distinct biochemical routes may therefore be differentially influenced by glycolytic flux, lactyl-CoA availability, methylglyoxal metabolism, cellular redox conditions, and subcellular localization (34–38).

These two mechanisms should not, however, be viewed as mutually exclusive. Their relative contributions to cellular Kla may vary according to cell type, metabolic state, subcellular environment, and pathological context. Although individual studies have emphasized enzymatic or non-enzymatic mechanisms in different disease contexts, current evidence does not establish that either pathway is consistently dominant across a given disease context. This issue is particularly unresolved in neutrophils, for which direct quantitative comparisons of the two routes remain limited. Moreover, changes in global Kla cannot by themselves distinguish between enzyme-mediated and non-enzymatic lactylation.

Clarifying the relative contributions of these pathways will require approaches that directly interrogate their biochemical origins, including site-specific quantitative mass spectrometry, metabolic tracing, stereoisomer-resolved analysis, and selective perturbation of candidate lactyltransferases or metabolic pathways. Importantly, these approaches should be interpreted in combination, as no single method is sufficient to definitively assign a Kla event to an enzymatic or non-enzymatic route. Such studies will be important for determining whether specific Kla events in neutrophils primarily reflect regulated enzymatic modification, metabolite-driven chemical acylation, or a combination of both.

### 3.2 Delactylases as erasers: established functions and controversial reverse activity

Removal of lactyl groups is an important mechanism contributing to the dynamic regulation of lysine lactylation (Kla). Both zinc-dependent histone deacetylases (HDACs) and NAD^+^-dependent sirtuins (SIRTs) have been reported to possess delactylase activity (39–45). Among them, HDAC1–3 have been characterized as histone delactylases, whereas SIRT1, SIRT2, and SIRT3 have been reported to remove lactyl groups from specific histone or non-histone substrates in distinct experimental systems (39–45). These findings support the involvement of HDACs and SIRTs in the enzymatic regulation of Kla turnover, although their substrate specificities and relative contributions may vary across cellular and metabolic contexts.

An additional layer of complexity has emerged from recent evidence suggesting that Class I HDACs may also catalyze lysine lactylation. A recent biochemical and cellular study reported that HDAC1–3 can catalyze lactylation and proposed that this reverse reaction may contribute substantially to Kla formation (46). In contrast, earlier biochemical studies established HDAC1–3 as efficient delactylases, with HDAC1–3 showing robust activity toward lactylated lysine substrates *in vitro* and HDAC1 and HDAC3 exhibiting delactylase activity in cells (36, 47, 48). Thus, Class I HDACs have now been reported to possess both delactylase and lactyltransferase activities, although the relative contribution and physiological relevance of these opposing reactions remain unresolved.

More broadly, current evidence supports the involvement of HDACs and SIRTs in the enzymatic removal of lactyl groups, but the substrate specificity, cellular functions, and physiological relevance of individual delactylases remain incompletely defined. Notably, most of the biochemical and cellular evidence establishing delactylase activity has been obtained from non-neutrophil systems, and direct validation of individual delactylases in primary neutrophils remains limited. Further studies are therefore needed to determine whether and how specific delactylases regulate Kla dynamics and neutrophil functional responses under physiological and pathological conditions.

### 3.3 Readers and the competitive PTM landscape

The biological consequences of Kla may, in principle, be influenced by proteins that recognize lactylated lysine residues. Among the best-characterized acylation readers, YEATS-domain proteins, including ENL, AF9, and YEATS2, recognize multiple lysine acylation states and have been particularly well characterized as readers of histone crotonylation (49–55). Structural and biochemical studies have demonstrated that the YEATS domains of AF9 and related proteins can directly engage crotonylated histone peptides, with AF9 showing a preference for crotonyl- over acetyl-lysine in several histone contexts (52–54).

However, these findings should not be interpreted as evidence that YEATS proteins are established readers of Kla. Current structural evidence has primarily defined YEATS-mediated recognition of crotonylation and other acyl-lysine modifications, whereas direct and quantitative evidence for specific YEATS-domain recognition of Kla remains limited. Indeed, the preferential recognition of Kcr by several YEATS domains indicates that the cellular interpretation of Kla may depend not only on the presence of lactylated residues but also on the relative abundance, accessibility, and chemical properties of competing acylation marks (49–55). AF9 YEATS, for example, contains an aromatic recognition pocket that preferentially accommodates crotonylated lysine, providing a structural basis for competition among chemically related acyl-lysine modifications.

Lactylation therefore occurs within a broader and dynamic lysine post-translational modification landscape that includes acetylation, crotonylation, succinylation, ubiquitination, and other modifications (30, 37). These modifications may occur on overlapping lysine residues and can influence protein structure, stability, chromatin accessibility, and transcriptional activity. Consequently, the functional effects of Kla should be considered in the context of other coexisting lysine modifications rather than as an isolated epigenetic signal.

This competitive landscape may be particularly relevant to metabolically active neutrophils. Metabolic changes may alter the relative availability of acyl-CoA substrates, thereby influencing the formation and distribution of different lysine acylation marks. In turn, the abundance, accessibility, and relative recognition of these modifications by reader proteins may shape their biological effects. However, direct quantitative evidence defining the extent to which such metabolic and epigenetic competition occurs in primary neutrophils remains limited. Thus, the interplay between lactylation and other lysine acylation pathways should be regarded as a potential regulatory mechanism that requires further experimental validation in neutrophils.

## 4 Regulatory roles of lysine lactylation in neutrophil functional remodeling

Neutrophils exhibit substantial functional and transcriptional heterogeneity and can adopt context-dependent states in response to inflammatory cues, tissue signals, hypoxia, and metabolic conditions (56–58). Emerging studies have linked Kla to several aspects of neutrophil biology, including migration, inflammatory activation, NET formation, and context-dependent functional remodeling (59–65). However, the strength of evidence varies considerably among these processes. Some studies directly examine lactylation in primary neutrophils, whereas others rely on tissue-level measurements, other cell types, indirect molecular markers, or lactylation-related gene signatures. This distinction is important when evaluating the contribution of Kla to neutrophil functional remodeling.

### 4.1 Lactylation-associated regulation of neutrophil migration and tissue infiltration

Neutrophil recruitment and tissue infiltration are essential components of innate immune responses and are regulated by inflammatory, metabolic, and tissue-derived signals. Several studies have linked lactate metabolism or lactylation-associated pathways to neutrophil recruitment in both malignant and non-malignant conditions.

In pancreatic cancer, histone H3 lysine 18 lactylation (H3K18la) in tumor cells has been associated with increased CXCL1 expression and enhanced neutrophil infiltration (59). In contrast, an integrated lactylation-related analysis of myocardial infarction datasets identified associations between lactylation-related metabolic programs and neutrophil infiltration, although the cellular source and functional significance of these lactylation changes were not directly established (66).

More direct experimental evidence has linked lactylation-associated signaling to neutrophil inflammatory responses and migration. In a sleep-deprivation model, modulation of the ROS–H3K18la regulatory relationship was reported to be associated with neutrophilic inflammation (67). In a microplastic-exposure model, a ROS–histone lactylation pathway in neutrophils was associated with enhanced neutrophil migration (60). These findings suggest that lactylation-related mechanisms may contribute to neutrophil recruitment and inflammatory trafficking, although the specific lactylated substrates and causal relationships may differ across pathological contexts.

More direct evidence comes from myocardial ischemia/reperfusion injury. Wang et al. identified lysine 26 lactylation of S100A9 (S100A9-K26la) in neutrophils and demonstrated increased S100A9- K26la in both experimental myocardial ischemia/reperfusion injury and patients with acute myocardial infarction (61). Genetic manipulation of S100A9-K26la in mice showed that this modification promotes S100A9 nuclear translocation and the expression of migration-related genes, thereby enhancing neutrophil trafficking and cardiac recruitment. Among currently available studies, this work provides comparatively direct evidence linking a defined non-histone lactylation event within neutrophils to a specific migratory phenotype.

### 4.2 Lactylation and neutrophil inflammatory activation

Evidence from primary neutrophils supports a role for histone lactylation in inflammatory activation. In an ex vivo study using human neutrophils, LPS stimulation increased intracellular lactate and histone lactylation, particularly H4K8la, and was associated with enhanced cytokine release, ROS production, and chemotaxis (63). Mechanistically, H4K8la enrichment at the WTAP promoter increased WTAP expression, which enhanced m6A modification and stability of TLR2 mRNA, thereby amplifying inflammatory responses (63). These findings were further supported by experiments in septic mice and patients, providing relatively direct evidence for an H4K8la–WTAP–TLR2 regulatory pathway in neutrophil inflammatory activation (63).

This study provides a relatively strong example of neutrophil-specific evidence linking metabolic changes, histone lactylation, and functional responses. Nevertheless, the broader relevance of this pathway across different inflammatory conditions remains to be determined.

### 4.3 Lactylation and NET formation

Evidence linking Kla to NET formation remains limited and is derived from distinct cellular and experimental contexts. In a model of sepsis-associated acute kidney injury, increased H3K18 lactylation and PAD4 expression were associated with enhanced NET formation, and pharmacological inhibition of histone lactylation or PAD4 attenuated NETosis and tissue injury (62). However, the mechanistic experiments were performed primarily in kidney tissue and NRK-52E renal epithelial cells, rather than in isolated primary neutrophils. Although additional experiments examined lactate-treated neutrophils, these findings do not establish a neutrophil-intrinsic H3K18la–PAD4 axis as the primary mechanism underlying NET formation.

Evidence from another study suggests that lactylation may regulate NET formation indirectly through intercellular communication. In sepsis-associated acute kidney injury, lactate-induced HMGB1 lactylation in macrophages promoted the release of HMGB1-containing extracellular vesicles, which were subsequently taken up by neutrophils and activated cGAS–STING signaling to promote mitochondrial DNA release and NET formation (68). Thus, the lactylation event in this model occurs in macrophages rather than neutrophils, supporting an indirect macrophage–neutrophil mechanism rather than neutrophil-intrinsic lactylation.

Taken together, current evidence supports a potential role for lactylation-associated pathways in NET formation, particularly in sepsis-related tissue injury, but the underlying mechanisms differ substantially between models. Compared with the relatively direct evidence linking neutrophil S100A9-K26la to migratory activity, evidence for a causal role of neutrophil-intrinsic Kla in NET formation remains limited. Further studies using primary neutrophils and site-specific lactylation approaches are needed to determine whether Kla directly regulates the molecular machinery of NET formation.

### 4.4 Tumor-associated neutrophils and immunosuppressive remodeling

Tumor-associated neutrophils exhibit substantial functional heterogeneity and can adopt distinct functional states depending on the tumor microenvironment (56–58). Recent studies have linked lactylation-associated pathways to immunosuppressive neutrophil states.

In a KRASG12C-mutant colorectal cancer liver metastasis model, AKR1B10-associated histone lactylation was reported to promote PD-L1 expression in tumor-infiltrating neutrophils and suppress CD8^+^ T-cell activity (64). In glioblastoma, hypoxia increased glucose metabolism and lactate production in a CD71^+^ neutrophil subset, while histone lactylation was associated with ARG1 expression and T-cell suppression. Pharmacological interference with histone lactylation reduced the immunosuppressive activity of these neutrophils and delayed tumor progression in experimental models (65).

Lactylation-related regulation has also been implicated in polymorphonuclear myeloid-derived suppressor cells (PMN-MDSCs). In a murine colorectal cancer model, MNDA-associated H3K18 lactylation was linked to PMN-MDSC infiltration and immunosuppressive activity, with consequences for response to anti-PD-1 therapy (69). These findings suggest that lactylation-associated mechanisms may contribute to the functional remodeling of tumor-associated neutrophil populations. However, the specific lactylated substrates, the relative contributions of histone and non-histone lactylation, and the extent to which these mechanisms are conserved across human tumor-associated neutrophil subsets remain to be determined.

Importantly, lactylation should not be regarded as a universal determinant of neutrophil phenotype. Tumor-associated neutrophils, PMN-MDSCs, low-density neutrophils, and other neutrophil states can arise under distinct microenvironmental and developmental conditions. Whether Kla represents a shared regulatory mechanism across these populations or instead acts in a subset- and context-dependent manner remains an important question.

### 4.5 Evidence strength and unresolved mechanisms

Taken together, current studies provide growing evidence that lactylation-associated mechanisms may contribute to neutrophil functional remodeling, but the strength of mechanistic evidence varies substantially across biological processes. The most direct evidence comes from studies identifying a defined lactylated substrate in primary neutrophils and linking it experimentally to a specific phenotype, such as S100A9-K26la in neutrophil migration (61). The H4K8la–WTAP–TLR2 pathway likewise provides relatively direct evidence linking histone lactylation to inflammatory activation in primary neutrophils (63).

By contrast, evidence linking lactylation to NET formation remains more heterogeneous, including tissue-level observations and intercellular mechanisms in which the relevant lactylation event occurs in macrophages rather than neutrophils (62, 68). Similarly, lactylation-related gene signatures and tissue-level associations with neutrophil infiltration or disease outcomes do not by themselves demonstrate increased Kla within neutrophils or establish causality (59, 64, 66, 69).

Thus, elevated lactate levels, altered expression of lactylation-related genes, or increased global Kla should not be interpreted as evidence of a specific lactylation-dependent mechanism in neutrophils. Future studies combining site-specific quantitative Kla profiling, metabolic tracing, neutrophil-specific perturbation, and functional validation in primary human neutrophils and relevant *in vivo* models will be important for defining the causal and context-dependent roles of lactylation in neutrophil biology.

## 5 Translational perspectives and therapeutic opportunities

The emerging links between lactate metabolism, lysine lactylation (Kla), and neutrophil function have highlighted lactylation-associated pathways as potential therapeutic targets in inflammatory and malignant diseases. Current strategies can be broadly categorized into three approaches: modulating lactylation-associated enzymes, targeting upstream metabolic pathways that influence lactate availability, and interfering with downstream effectors of lactylation. However, most evidence remains preclinical, and few interventions have been shown to selectively modulate lactylation in neutrophils. Therefore, the therapeutic potential of targeting lactylation-associated pathways should be interpreted cautiously, particularly given the broad metabolic and immunological functions of these pathways across different cell types.

### 5.1 Pharmacological targeting of lactylation-associated pathways

Inhibition of p300/CBP represents one potential strategy for reducing enzyme-mediated lactylation. However, p300/CBP catalyzes multiple lysine acylation reactions and regulates diverse transcriptional programs, and currently available p300 inhibitors are not selective for lactylation. Experimental studies in cancer models have demonstrated antitumor effects of targeting p300-associated pathways (70), but direct evidence that these effects result from suppression of neutrophil lactylation remains limited. Therefore, p300 inhibition should currently be viewed as a broader epigenetic intervention rather than a Kla-specific therapeutic strategy.

Metabolic modulation represents another potential approach. Urolithin A has been reported to attenuate neutrophil-associated inflammation in a sleep-deprivation model, accompanied by reduced lactate accumulation and suppression of the ROS–H3K18la pathway (67). These findings suggest that metabolic interventions can influence lactylation-associated inflammatory responses. However, Urolithin A affects multiple metabolic and signaling pathways and should not be considered a selective lactylation inhibitor.

More targeted evidence has emerged from tumor and ischemic injury models. In glioblastoma, isosafrole was reported to interfere with hypoxia-driven histone lactylation in tumor-infiltrating neutrophils, reducing ARG1-associated immunosuppressive activity and delaying tumor progression in experimental models (65). In myocardial ischemia/reperfusion injury, α-lipoic acid inhibited DLAT-mediated S100A9-K26la and reduced neutrophil recruitment and cardiac inflammation (61). These findings provide proof-of-concept that lactylation-associated pathways can be pharmacologically modulated, but the specificity, pharmacokinetics, systemic effects, and long-term safety of these compounds remain insufficiently characterized.

Overall, current pharmacological approaches should be interpreted as lactylation-associated or metabolism-modulating interventions rather than clinically validated Kla-specific therapies. This distinction is particularly important because broad modulation of glycolysis, p300/CBP activity, or other metabolic pathways may affect physiological neutrophil functions, including antimicrobial defense, phagocytosis, and tissue repair.

### 5.2 Lactylation-associated biomarkers

Lactylation-associated biomarkers can be broadly categorized into direct measurements of lactylated proteins and indirect molecular signatures associated with lactylation-related pathways. Direct candidates include site-specific modifications such as S100A9-K26la and H4K5la, whereas lactylation-related gene signatures derived from transcriptomic or computational analyses represent indirect indicators of lactylation-associated cellular states.

S100A9-K26la provides a relatively direct example of a candidate neutrophil-associated lactylation biomarker. Increased S100A9-K26la was detected in neutrophils from patients with acute myocardial infarction and was associated with myocardial inflammatory injury in experimental MI/R models (61). These findings support further investigation of site-specific lactylation as a potential biomarker of neutrophil activity and disease-associated inflammation.

Histone lactylation has also been investigated in sepsis. Increased H4K5la in peripheral neutrophils was associated with inflammatory markers and disease-severity indices, including C-reactive protein (CRP), procalcitonin (PCT), and Sequential Organ Failure Assessment (SOFA) scores (71). These findings suggest that neutrophil-associated H4K5la may have potential value for disease assessment or risk stratification, although its clinical utility requires validation in larger, prospective cohorts.

By contrast, lactylation-related gene signatures derived from bulk transcriptomic datasets should be interpreted more cautiously. Such signatures may correlate with immune infiltration, prognosis, or disease subtype (72–76), but gene expression does not directly measure Kla and cannot establish that the inferred lactylation-associated state occurs within neutrophils. Similarly, CBR1 and other lactylation-associated genes may reflect disease-related metabolic states but should not be classified as direct lactylation biomarkers without direct evidence of altered Kla.

Future biomarker studies should therefore prioritize quantitative, site-specific measurements of Kla, together with assessment of diagnostic or prognostic performance in prospective, clinically well-characterized cohorts.

### 5.3 Challenges for clinical translation

Despite encouraging preclinical findings, several barriers currently limit the clinical translation of lactylation-associated interventions. First, no established therapy currently selectively targets neutrophil lactylation. Second, most available compounds affect multiple metabolic or epigenetic pathways, making it difficult to attribute their effects specifically to Kla. Third, systemic modulation of glycolysis, lactate metabolism, p300/CBP, or related pathways may interfere with physiological immune functions and tissue homeostasis.

These considerations are particularly important for neutrophil-directed therapies because neutrophils are essential for antimicrobial defense. Although excessive neutrophil activation may contribute to pathological inflammation, broad suppression of neutrophil function could also compromise host defense. Therapeutic strategies will therefore need to balance attenuation of pathological neutrophil responses with preservation of essential immune functions.

Neutrophil heterogeneity further complicates clinical translation. Tumor-associated neutrophils, PMN-MDSCs, low-density neutrophils, and other functionally distinct populations may respond differently to metabolic and lactylation-associated signals. Selective targeting of disease-associated neutrophil states, rather than broad suppression of neutrophil activity, may therefore represent a more feasible therapeutic strategy. However, this concept remains largely experimental and requires validation in clinically relevant models.

## 6 Conclusion and perspectives

Lysine lactylation has emerged as a potential molecular link between cellular metabolism and neutrophil function. Growing evidence suggests that lactylation-associated mechanisms may contribute to neutrophil migration, inflammatory activation, NET formation, and tumor-associated functional remodeling. However, the strength of evidence varies substantially across these biological processes. While several studies have identified defined lactylated proteins or histone marks in neutrophils and linked them to specific functional outcomes, other findings derive from tissue-level analyses, other cell types, or lactylation-associated molecular signatures and therefore do not necessarily establish neutrophil-intrinsic mechanisms.

Several fundamental questions remain unresolved. The relative contributions of enzymatic and non-enzymatic lactylation to global Kla accumulation have not been quantitatively defined in neutrophils or across pathological contexts. The proposed lactyltransferase activity of HDAC1–3 remains controversial and lacks definitive *in vivo* validation in neutrophils. In addition, the relationships among extracellular lactate signaling, metabolic substrate availability, competing lysine acylations, and lactylation reader recognition require further investigation. These uncertainties underscore the importance of distinguishing established mechanisms from emerging or speculative models.

Methodological limitations also remain an important barrier to the field. Improved site-specific quantitative mass spectrometry, metabolic tracing, stereochemistry-resolved analysis, and more specific analytical reagents will be necessary to define the biochemical origin and functional significance of individual Kla events. Future studies should prioritize validation in primary human neutrophils and relevant *in vivo* models while accounting for neutrophil heterogeneity, including tumor-associated neutrophils, PMN-MDSCs, low-density neutrophils, and other context-dependent populations.

From a translational perspective, targeting lactate metabolism or lactylation-associated pathways represents a promising but still preclinical strategy. Most currently available compounds affect multiple metabolic or epigenetic processes and cannot yet be considered selective Kla-targeting agents. Their specificity, off-target effects, systemic safety, and potential interference with essential neutrophil-mediated host defense require careful evaluation. Overall, current evidence supports lactylation as an important emerging mechanism linking metabolism to neutrophil biology, but further mechanistic, quantitative, and translational studies are needed to establish its precise contribution and therapeutic relevance.
